# Supplementary material for: Somatic structural variants drive distinct modes of oncogenesis in melanoma
Source: J Clin Invest. 2024 May 14;134(13):e177270. doi: 10.1172/JCI177270 (PMC11213511; doi:10.1172/JCI177270)
Supplement: Supplemental data [file jci-134-177270-s166.pdf]

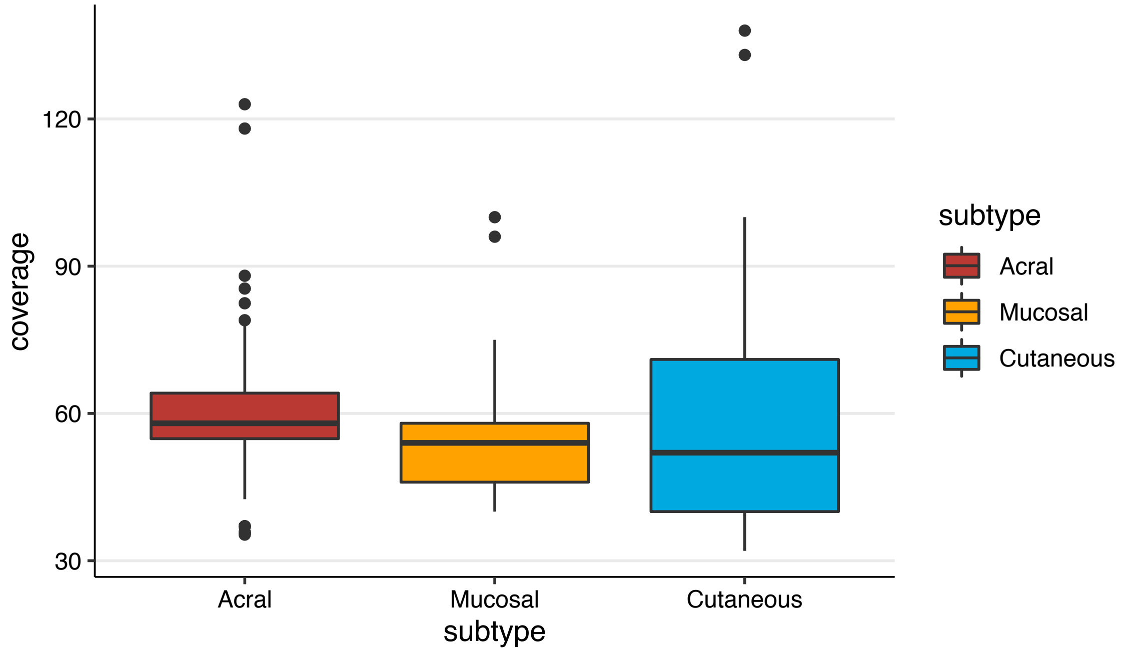

Supplementary Figure 1: Distribution of sequencing coverage by histological subtype
There was no difference in the sequencing coverage between samples from different histological subtypes (Wilcoxon-Mann-Whitney, $p = 0.08$).

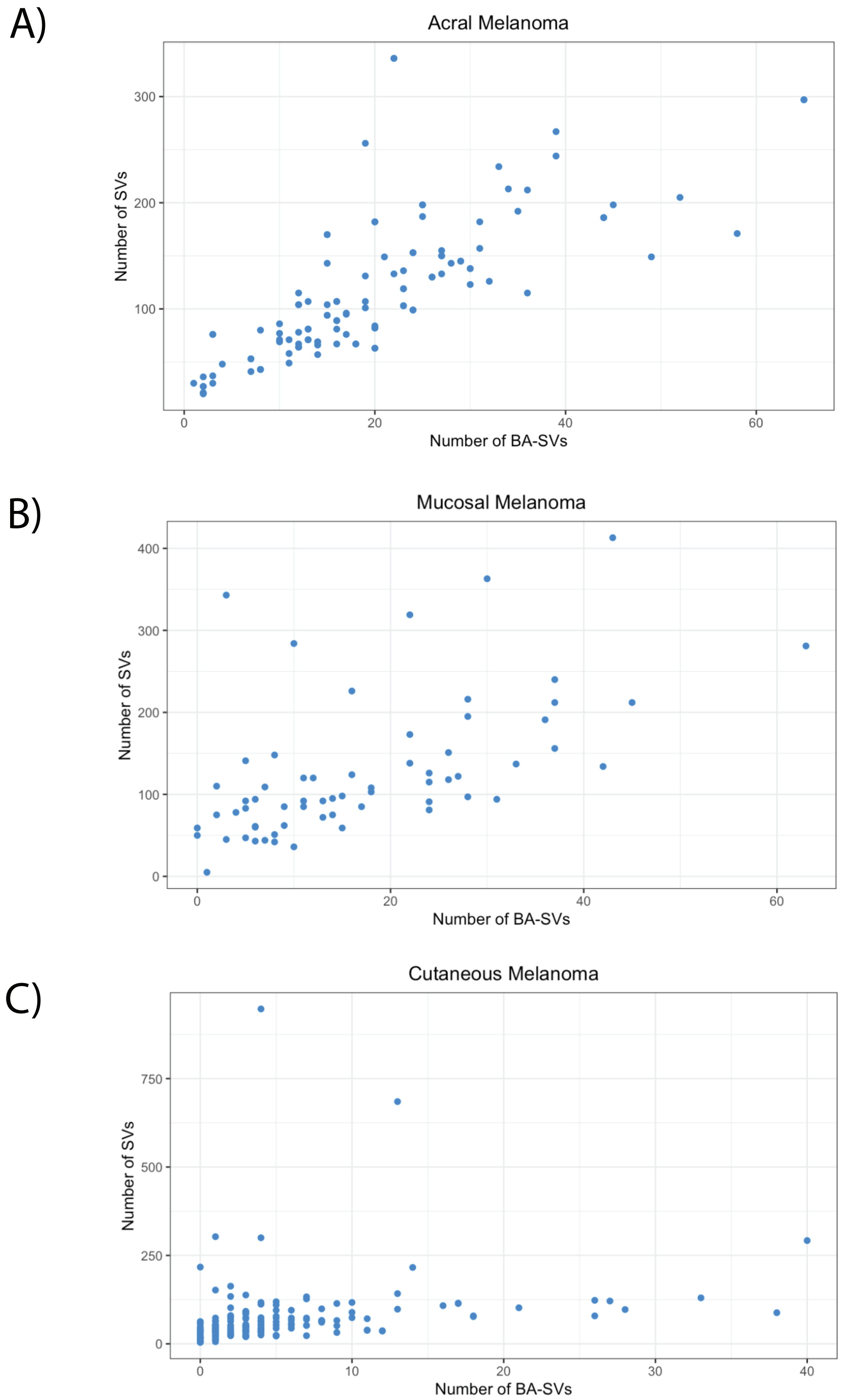

Supplementary Figure 2: SV burden associations with BA-SV frequency
(a) There is a strong association between SV burden and BA-SV frequency in acral melanomas (Pearson's r = 0.75, p = 2.2×10^{-16}), compared to a **(b)** moderate association in mucosal melanomas (Pearson's r = 0.57, p = 8.8×10^{-7}), and a **(c)** weak association in cutaneous melanomas (Pearson's r = 0.26, p = 6.2×10^{-4}).

A)

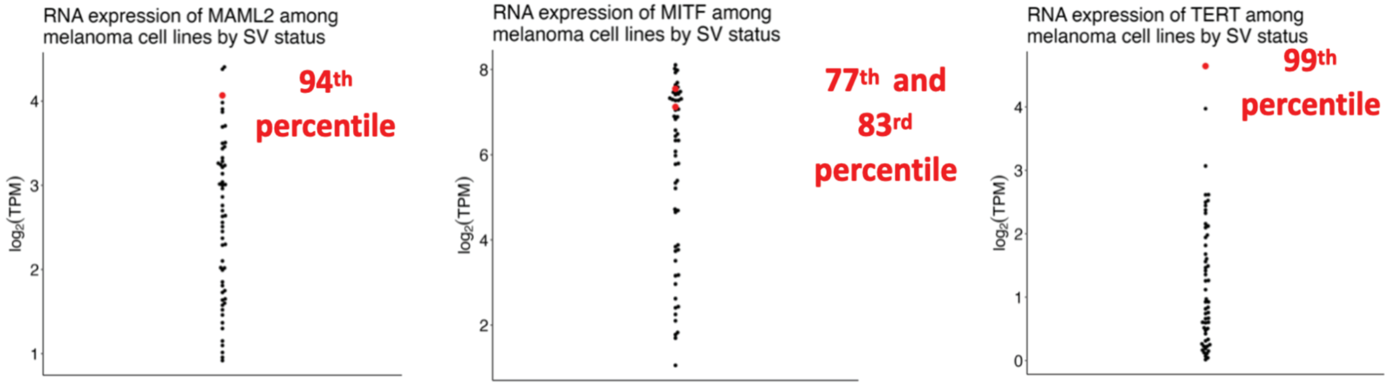

B)

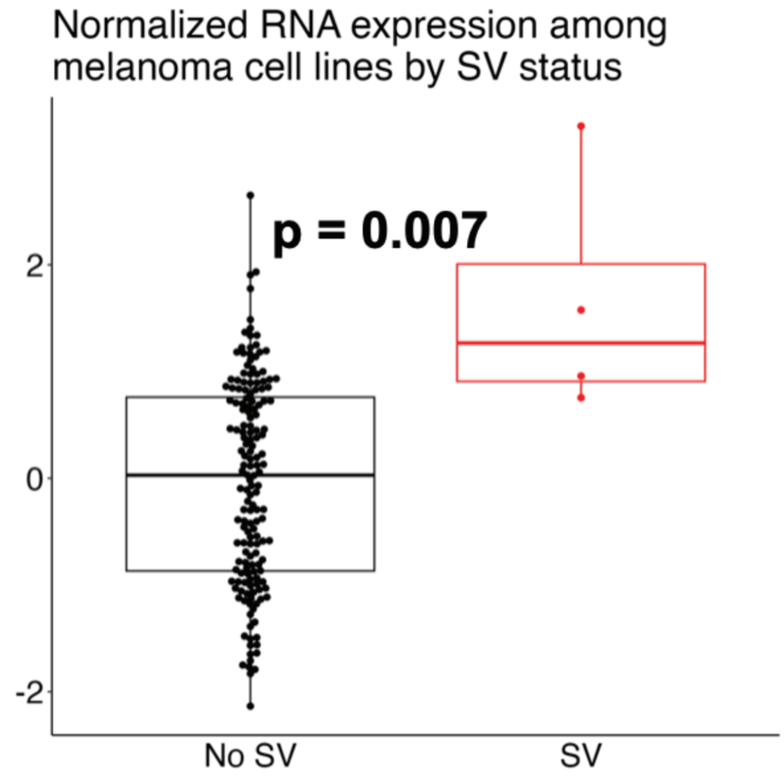

Supplementary Figure 3: Functional consequences of highlighted and overlapping SVs in CCLE (a) Percentile gene expressions of cell lines with SVs affecting genes highlighted in Figure 3. **(b)** Boxplot showing the normalized expression of genes affected by SVs compared to those that are unaffected.The p-value is from performing a Mann-Whitney test on the pan-gene aggregated z-scored (i.e. each gene z-scored separately to put the expression values on the same scale) expression.

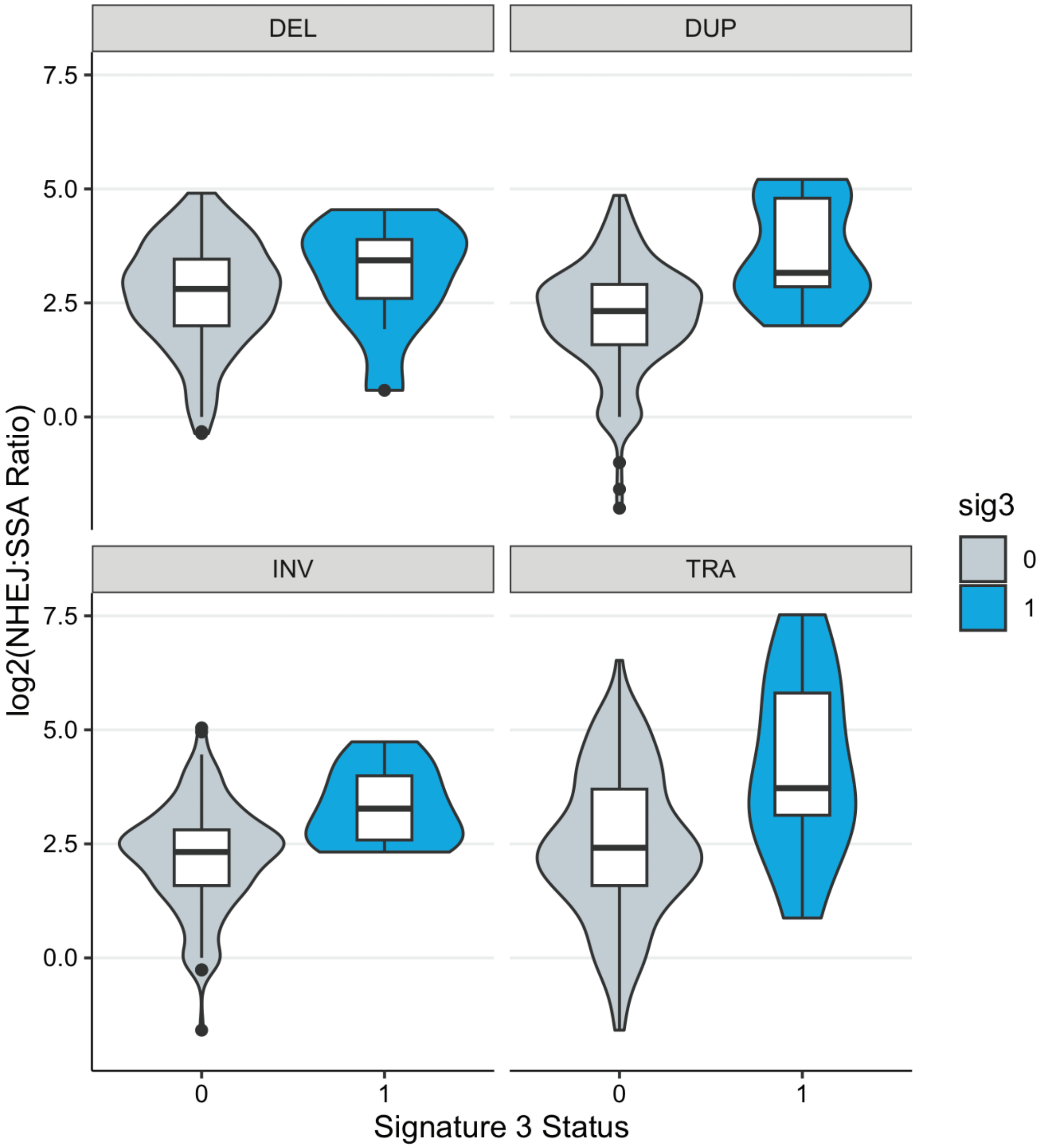

Supplementary Figure 4: Distribution of NHEJ:SSA ratio between signature 3 and non-signature 3 tumors by SV type

The difference between the NHEJ:SSA ratio in SBS3-positive and negative tumors was statistically significant and equally as enriched in SBS3 positive tumors across DUP, INV, and TRA events (Mann-Whitney U, $p < 6.9 \times 10^{-3}$), but not DEL events (Mann-Whitney U, $p > 0.05$).

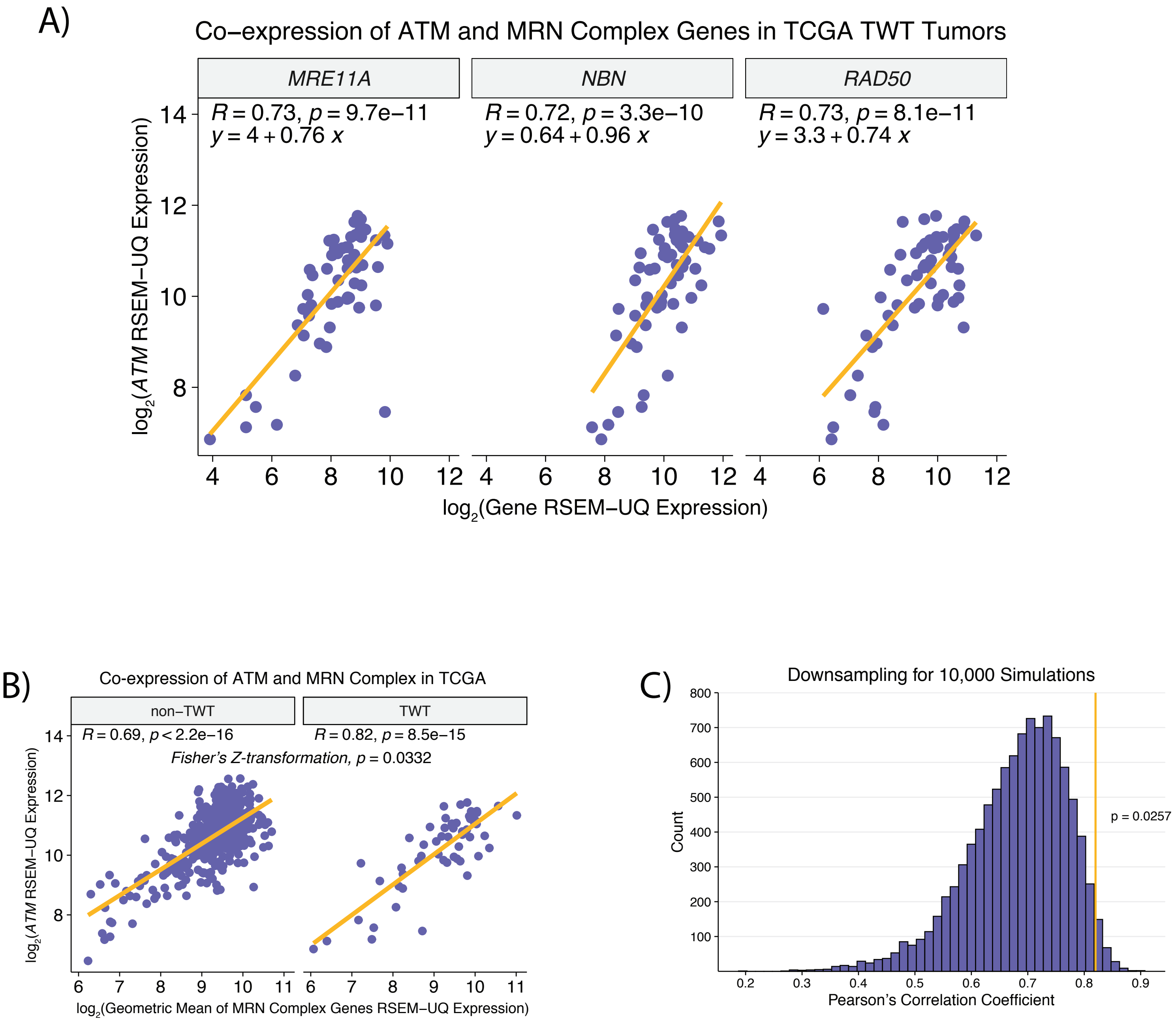

Supplementary Figure 5: Association between *ATM* and the MRN complex in TWT and non-TWT Melanomas

(a) No difference was observed in the association between *MRE11A* or *NBN* expression and *ATM* expression compared to the association between *RAD50* and *ATM* expression in TWT tumors **(b)** The correlation between *ATM* expression and MRN complex expression (methods) in non-TWT and TWT cutaneous melanoma tumors. **(c)** The distribution of Pearson's correlation coefficients from 10,000 randomly sampled simulations where non-TWT cutaneous tumors are downsampled to the number of TWT cutaneous tumors in the cohort.

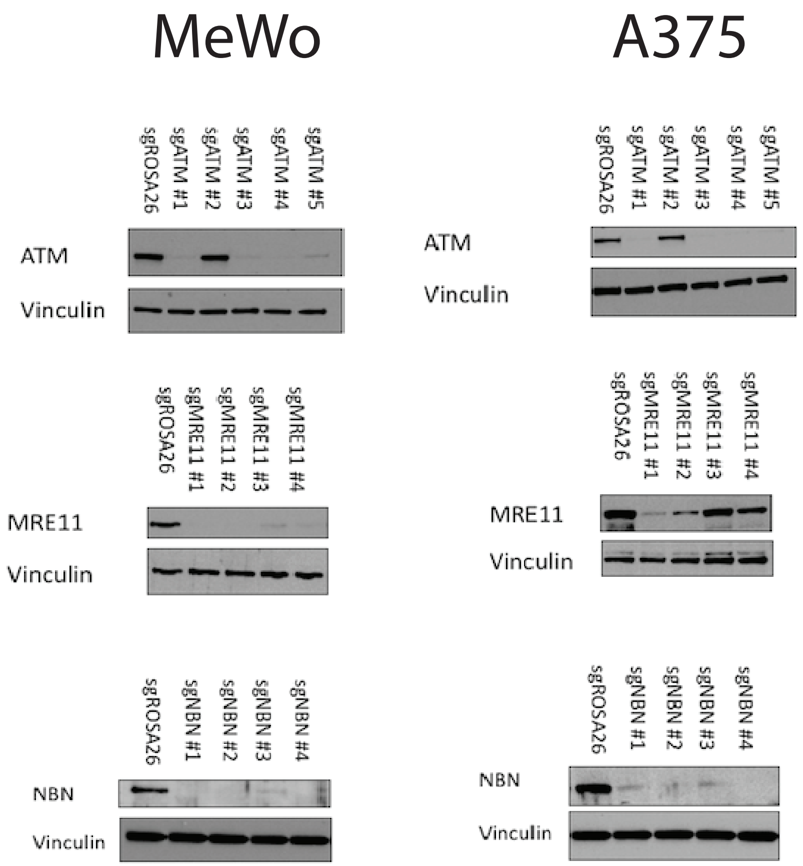

Supplementary Figure 6: Western Blots for MeWo and A375 melanoma cell lines
Western blots for the MeWo and A375 melanoma cell lines for knockouts of *ATM*, *MRE11*, and *NBN*. Vinculin is used a negative control.
